# Subendometrial resistence and pulsatility index assessment of endometrial receptivity in assisted reproductive technology cycles

**DOI:** 10.1186/s12958-019-0507-6

**Published:** 2019-08-02

**Authors:** R. Silva Martins, A. Helio Oliani, D. Vaz Oliani, J. Martinez de Oliveira

**Affiliations:** 1 Centro Hospitalar Universitário Cova da Beira EPE, Quinta do Alvito, 6200 503 Covilha, Portugal; 2 Centro Investigação Ciências da Saúde – Faculdade Ciências da Saúde, Universidade da Beira Interior, Alameda Infante D, Henrique, 6200 506 Covilha, Portugal

**Keywords:** Endometrial receptivity, Assisted reproductive technology, Subendometrial blood flow, Uterine artery fluxometry, Embryo implantation

## Abstract

**Objective:**

To evaluate Subendometrial and Uterine artery resistance and pulsatility index continuous analysis as a predictor of Endometrial receptivity in Assisted Reproductive Technology (ART) Cycles.

**Design:**

Serial 2D transvaginal coloured power doppler ultrasound performed in women on ART cycle to evaluate a pattern that better predicts implantation rates. One hundred sixty-nine subjects on a prospective case control study were assessed. Uterine artery and Subendometrial resistance and pulsatility index was performed to all subjects at baseline (prior to ovarian controlled stimulation), at day 6, 8 and 10 of controlled ovarian stimulation, at trigger day and at embryo transfer day. Also the ratio of fluxometric parameters between Subendometrial blood flow and uterine artery was measured.

**Results:**

No statistical difference was noted between two groups in terms of demographics and ART procedures and scores. Uterine artery resistance and pulsatility index showed statistical difference between the two groups (implantation versus non-implantation group). Also statistical significance was obtained between two groups in terms of Subendometrial vascularization. Ratio between Subendometrial and Uterine artery showed lower values of fluxometric parameters in all range for the Subendometrial territory.

**Conclusions:**

Serial Subendometrial and Uterine artery fluxometry may be a useful tool for clinicians in predicting endometrial receptivity enhancing elective embryo transfers in the same ART cycle.

## Introduction

Human implantation is a complex process requiring synchrony between a healthy embryo and a functionally competent or receptive endometrium [1]. Since the introduction of assisted conception, many techniques have been developed to further improve ovarian stimulation, oocyte retrieval, and embryo culture [2]. However there has always been a lack in understanding the endometrial characteristics compatible with a successful pregnancy. To prepare for pregnancy, the endometrial lining in the uterus thickens and becomes receptive to implantation of a fertilized egg. This happens in response to hormone secretion, with oestrogen and progesterone being the primary hormones that are released to ensure the endometrial lining is receptive to pregnancy [3]. Diagnosis of endometrial receptivity (ER) has posed a challenge and so far, most available tests have been subjective and lack accuracy and a predictive value. Microarray technology has allowed identification of the transcriptomic signature of the window of receptivity - window of implantation (WOI). This technology has led to the development of a molecular diagnostic tool, the ER array (ERA) for diagnosis of ER [4]. The ERA is a tissue test, which evaluates the receptivity of the endometrial lining to determine the window of implantation. It is performed based on the assumed WOI for a woman during a natural cycle or an HRT (hormone replacement therapy) cycle [5]. The test consists of an endometrial biopsy to determine the optimal timing for implantation in a round of Assisted Reproductive Technology (ART) cycle [6]. Nevertheless, ERA requires an invasive procedure, it has an associated cost, and in women with irregular cycles may not prove to be cost-efficient. Ultrasound is a non-invasive technique that can assess changes in the endometrium during stimulated cycles. The use of high-resolution transvaginal probes made possible follow up throughout the cycle of endometrium changes [7]. Uterine receptivity is regulated by a number of factors including uterine perfusion, and better yet endometrial perfusion. Differences between infertile and fertile women uterine perfusion have been reported. It has been suggested that impaired uterine and endometrial perfusion may be the cause of failure. In ART cycles blood flow resistance in uterine artery and in the endometrial territory has been reported to be a predictive indicator of implantation. However using this method is still controversial in clinical applications, and many studies reported a small sample size or a single one moment observation. From a clinical point of view some objective parameters must be obtained in order to ascertain the likelihood of an on going pregnancy in ART cycles, preferably in a non-invasive and cost efficient way [8]. Many studies have been conducted on the hemodynamic changes of utero-ovarian arteries during ART cycles. For optimizing the results of ART it is critical to decide the best timing for embryo transfer. With the introduction of high-resolution transvaginal probes, the non-invasive, accessible transvaginal sonography made it particularly suitable for serial follow up throughout the stimulated cycle. The aim of this prospective study is to further evaluate the capability of serial measurement of subendometrial fluxometry dopppler flow as a non-invasive procedure to determine endometrial receptivity [9–11].

## Material and methods

Prospective case control study of 169 women in ART cycles. All data collected and informed written consent was obtain according to the Ethics Committee of our Institution.

Only subjects with viable good grade embryos for transfer (double embryo transfer on day 3 of development) were selected. All subjects have been in a short protocol regimen with antagonist for ovarian stimulation using gonadotropins. All used recombinant human gonadochorionic hormone (rhCG) for induction of ovulation 36 h prior to oocyte pick up. Demographics data was collected for all patients and serial ultrasound analysis (uterine artery fluxometry and subendometrial blood blow) was performed using the same protocol for all participating subjects. Colour Doppler signals are measured in uterine artery and their ascending branches located in the outer third of the myometrium. The impedance of blood flow through the uterine arteries may be expressed as the pulsatility index (PI), unit less and angle independent, and the resistance index (RI), unit less and angle dependent. The PI is measured from the flow velocity waveforms as the systolic peak velocity minus end-diastolic velocity divided by the mean. It can be classified as low (0.00–1.99), medium (2.00–2.99) and high (over 3.0). The RI is calculated as the ratio of peak systolic flow velocity minus end-diastolic velocity divided by peak systolic velocity, ranging from 0.0 to 1.0. Subendometrial blood flow represents vessels irrigating endometrium within 10 mm of the lateral endometrium border [12–15]. During ovarian controlled stimulation serial ultrasound exams were performed and serum estradiol levels obtained for all participants. Uterine and subendometrial resistance and pulsatility index was obtained with 2D sagittal uterine view with power coloured doppler in all evaluations (Basal moment – Day 2 or 3 of women menstrual cycle and prior to begin of ovarian controlled stimulation; at Day 6, Day 8 and Day 10 after the begin of ovarian controlled stimulation; at Trigger day; and at Embryo Transfer). Blood flow evaluations were performed in the morning to avoid fluctuation due to circadian rhythm of uterine artery blood flow [16–18].

At Day 12 after embryo transfer, human gonadochorionic sub-unit B serum levels were obtained, and groups were set: positive results (for values over 5 International Units - IU) and negative results (for values under 5 IU). All data collected was analysed between these two set groups and compared.

All data was analysed using SPSS (Statistical Package for Social Sciences) version 25.0. Results are presented as mean values with standard deviation (SD). Comparisons between means among the study groups were performed using independent samples t-Test. A Value of *p* < 0.05 was considered statistically significant.

The authors do not report any conflict of interest.

The study protocol has been approved by the Ethics Committee of our Institution (CHCB 22/2017), in accordance with the relevant guidelines and regulations.

## Results

Uterine artery resistency and pulsatility index, as well as subendometrial blood flow resistance and pulsatility index was obtained in all 169 cycles using 2D power doppler transvaginal ultrasound in continuous observations. Demographics characteristics and ART parameters are shown in Table 1. Women were divided into two groups depending on the value of hCG at Day 12 after embryo transfer: 123 in the negative group – non-implantation group (72.8%) and 46 on the positive group – implantation group (27.2%). There were no statistical difference between two set groups in terms of demographics and ART parameters.

**Table 1:** Demographics and assisted reproductive technology cycle parameters between two groups. (Implantation, *N* = 46 and Non-Implantation, *N* = 123). Descriptive statistics between two groups. Mean values with standard deviation (SD)

	Non-Implantation *N* = 123 (72.8%)	Implantation *N* = 46 (27.2%)	t-Test *p* value
Female Age (in years)	34.94 ± 4.03 (19–39)	34.28 ± 3.35 (25–39)	0.290
Male Age (in years)	36.14 ± 4.76 (22–46)	37.19 ± 5.91 (29–62)	0.832
Time of Infertility (in months)	54.46 ± 33.82 (12–204)	60.22 ± 38.49 (14–192)	0.375
Type of Infertility:			0.297
Primary	95/123 (77.2%)	38/46 (82.6%)	
Secondary	28/123 (22.8%)	8/46 (17.4%)	
AntiMullerian hormone (pg/mL)	2.45 ± 2.45 (0.09–16.65)	2.62 ± 2.46 (0.04–13.56)	0.679
Antral follicle count	8.43 ± 5.07 (2–40)	8.63 ± 3.74 (2–20)	0.801
Total dose of gonadotropins (in International Units)	2500.81 ± 812.19 (300–4500)	2508.15 ± 757.91 (450–4500)	0.956
Progesterone levels at Trigger day (ng/mL)	0.88 ± 0.44 (0.01–2.20)	0.78 ± 0.47 (0.01–2.10)	0.188
Number of collected Oocytes	8.25 ± 5.14 (2–22)	10.50 ± 5.20 (2–23)	0.140
Metaphase II Oocytes	6.57 ± 4.22 (2–17)	7.06 ± 4.77 (2–21)	0.150
Number of day 3 embryos	3.18 ± 2.40 (2–12)	3.84 ± 2.65 (2–12)	0.120
Number of blastocyst for vitrification	0.65 ± 1.51 (0–6)	0.86 ± 1.71 (0–9)	0.200

**Fig. 1:**
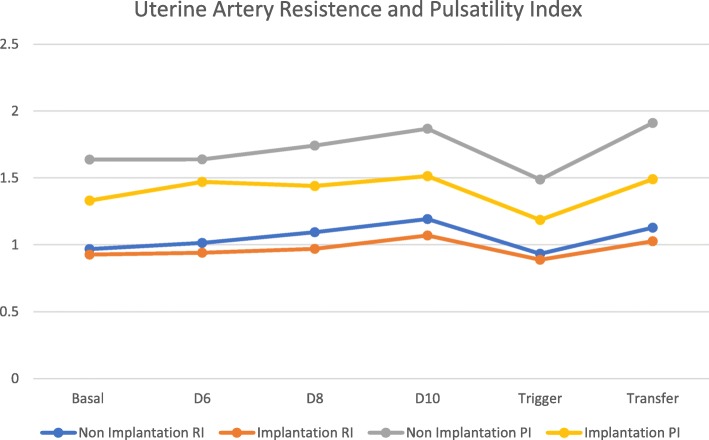
Serial Uterine Artery Resistance and Pulsatility index flow (Mean values). RI – Resistance Index; PI – Pulsatility Index

Uterine artery blood flow showed no statistical difference between two groups at baseline, both for resistance and pulsatility index. Statistical difference between two groups is shown after day 6 of ovarian controlled stimulation for both parameters in analysis (Table 2 and Fig. 1). We can see that both resistance and pulsatility index increase its values slightly until trigger day with hCG. The results are however; always lower for the implantation group. (trigger day with hCG 0.93 ± 0.10 on the non-implantation group versus 0.88 ± 0.09 for the implantation group with *p* value of 0.011 in the resistance index and 1.48 ± 0.38 versus 1.18 ± 0.27 with *p* value of 0.001 for the pulsatility index). After trigger day values tend to return to previously observed during controlled ovarian stimulation.

**Table 2:** Ultrasound parameters between two groups. (Uterine artery resistance index and uterine artery pulsatility index) at baseline, at day 6, 8 and 10 after controlled ovarian stimulation, at trigger day and at embryo transfer day. Mean values with standard deviation (SD). rhCG – recombinant Human chorionic gonadotropin

	Non-Implantation *N* = 123 (72.8%)	Implantation *N* = 46 (27.2%)	t Test *p* value
Uterine Resistance Index (Ut RI)			
Basal	0.97 ± 0.16	0.92 ± 0.12	0.1
Day 6	1.01 ± 0.15	0.94 ± 0.11	**0.04**
Day 8	1.09 ± 0.14	0.97 ± 0.12	**0.001**
Day 10	1.19 ± 0.16	1.07 ± 0.16	**0.001**
Trigger Day with rhCG	0.93 ± 0.10	0.88 ± 0.09	**0.011**
Embryo Transfer Day	1.12 ± 0.12	1.02 ± 0.09	**0.001**
Uterine Pulsatility Index (Ut PI)			
Basal	1.46 ± 0.51	1.33 ± 0.34	0,06
Day 6	1.64 ± 0.45	1.47 ± 0.40	**0,023**
Day 8	1.74 ± 0.47	1.44 ± 0.44	**0.001**
Day 10	1.87 ± 0.43	1.51 ± 0.37	**0.001**
Trigger Day with rhCG	1.48 ± 0.38	1.18 ± 0.27	**0.001**
Embryo Transfer Day	1.91 ± 0.54	1.49 ± 0.42	**0.001**

**Fig. 2:**
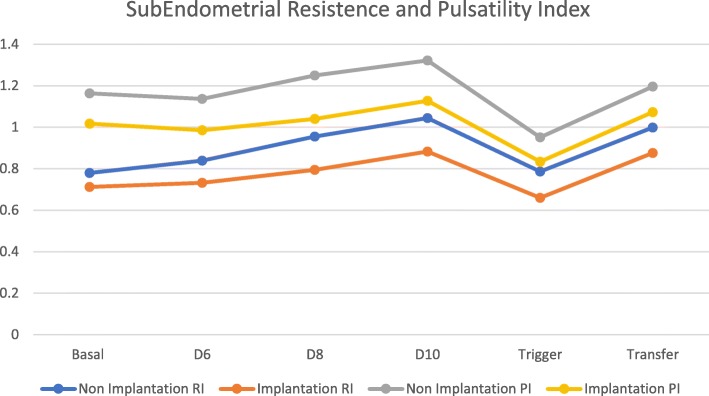
Serial Subendometrial artery Resistance and Pulsatility index flow (Mean values). RI – Resistance Index; PI – Pulsatility Index

Subendometrial blood flow analysis (resistance and pulsatility index) showed no statistical difference between two groups at baseline, with increasing values for both groups during controlled ovarian stimulation. During that period and also at trigger, and at embryo transfer day, there was statistical difference between two groups with lower scores for the implantation Group (resistance index of 0.78 ± 0.16 versus 0.65 ± 0.12 with *p* value of 0.001 and pulsatility index of 0.95 ± 0.14 versus 0.83 ± 0.14 with *p* value of 0.001 for non-implantation versus implantation group at trigger day respectively) (Table 3 and Fig. 2).

**Table 3:** Ultrasound parameters between two groups. (Subendometrial resistance index, subendometrial pulsatility index, subendometrial / uterine artery resistance index ratio and subendometrial / uterine artery pulsatility index ratio) at baseline, at day 6, 8 and 10 after controlled ovarian stimulation, at trigger day and at embryo transfer day. Mean values with standard deviation (SD). SE/Ut – Subendometrial / Uterine Arteries ratio; rhCG – recombinant Human chorionic gonadotropin

	Non Implantation *N* = 123 (72.8%)	Implantation *N* = 46 (27.2%)	t Test *p* value
Basal			
Subendometrial Resistance Index	0.77 ± 0.17	0.71 ± 0.17	0.82
Subendometrial Pulsatility Index	1.16 ± 0.25	1.01 ± 0.26	0.1
SE/Ut RI ratio	0.80 ± 0.09	0.76 ± 0.12	0.6
SE/Ut PI ratio	0.73 ± 0.12	0.77 ± 0.11	0.117
Day 6 after Controlled Ovarian Stimulation			
Subendometrial Resistance Index	0.84 ± 0.17	0.73 ± 0.14	**0.001**
Subendometrial Pulsatility Index	1.14 ± 0.20	0.98 ± 0.22	**0.001**
SE/Ut RI ratio	0.82 ± 0.10	0.77 ± 0.09	**0.04**
SE/Ut PI ratio	0.72 ± 0.14	0.68 ± 0.12	0.132
Day 8 after Controlled Ovarian Stimulation			
Subendometrial Resistance Index	0.95 ± 0.16	0.79 ± 0.18	**0.001**
Subendometrial Pulsatility Index	1.24 ± 0.20	1.03 ± 0.25	**0.001**
SE/Ut RI ratio	0.87 ± 0.13	0.81 ± 0.12	**0.04**
SE/Ut PI ratio	0.75 ± 0.15	0.73 ± 0.10	0.615
Day 10 after Controlled Ovarian Stimulation			
Subendometrial Resistance Index	1.04 ± 0.718	0.88 ± 0.19	**0.001**
Subendometrial Pulsatility Index	1.32 ± 0.23	1.12 ± 0.31	**0.001**
SE/Ut RI ratio	0.88 ± 0.11	0.82 ± 0.08	**0.04**
SE/Ut PI ratio	0.72 ± 0.10	0.75 ± 0.11	0.251
Trigger Day with rhCG			
Subendometrial Resistance Index	0.78 ± 0.16	0.65 ± 0.12	**0.001**
Subendometrial Pulsatility Index	0.95 ± 0.14	0.83 ± 0.14	**0.001**
SE/Ut RI ratio	0.84 ± 0.14	0.73 ± 0.09	**0.01**
SE/Ut PI ratio	0.72 ± 0.12	0.68 ± 0.11	**0.01**
Embryo Transfer Day			
Subendometrial Resistance Index	0.99 ± 0.15	0.87 ± 0.12	**0.001**
Subendometrial Pulsatility Index	1.19 ± 0.17	1.07 ± 0.20	**0.001**
SE/Ut RI ratio	0.84 ± 0.15	0.74 ± 0.10	**0.001**
SE/Ut PI ratio	0.66 ± 0.12	0.70 ± 0.08	**0.018**

**Fig. 3:**
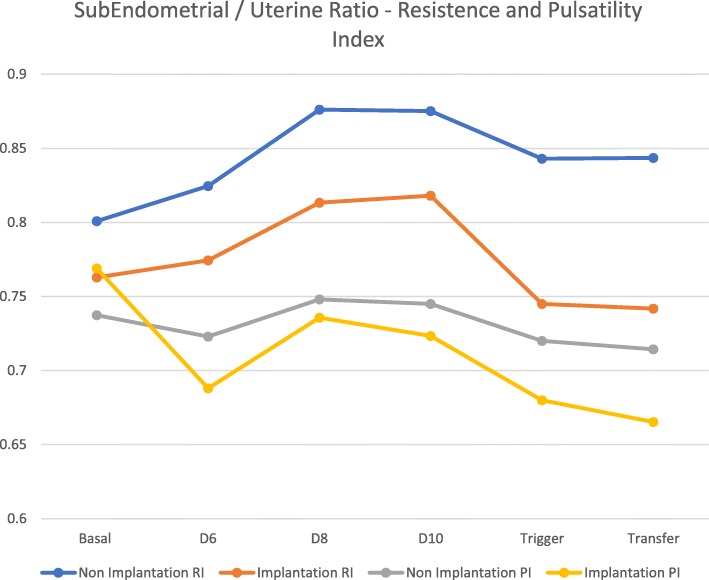
Serial Subendometrial / Uterine Arteries Ratio – Resistance and Pulsatility index flow (Mean values) RI – Resistance Index; PI – Pulsatility Index

The ratio between subendometrial blood flow and uterine artery fluxometry showed no statistical difference between both groups at baseline for the resistance index. Statistical difference between two groups was set after controlled ovarian stimulation, trigger day with hCG and at embryo transfer for the resistance Index. However, in terms of pulsatility index, no statistical difference was met between the two groups except for the trigger day with hCG and at embryo transfer day (0.72 ± 0.12 versus 0.68 ± 0.11 with *p* value of 0.01and 0.66 ± 0.12 versus 0.70 ± 0.08 with *p* value of 0.018, for the non-implantation versus implantation group respectively) (Fig. 3).

In this study the intraobserver reliability was 0.96. In addition, because the same operator performed all measurements, in this study there was no interobserver variability.

## Discussion

Endometrial receptivity in ART cycle has always been a challenge for physicians that need real time data in order to make better treatment options [12–15]. Vaginal 2D power doppler ultrasound is a non-invasive and a relative inexpensive tool at clinician’s disposal [16]. Single analysis of endometrial pattern at trigger day has been the most used, with contradictory findings. Also sample size of many studies led to conflicting results and further investigation in this area has been postponed with the advent of other technologies. In this study we aimed to address these issues in a wholesome way with several observations over time with a good sample size in order to obtain further data and better knowledge of the working process underlying endometrial receptivity.

Uterine artery fluxometry (resistance and pulsatility index) showed with statistical difference lower values in the implantation group in comparison to the non-implantation group. We could also monitor increasingly higher levels during controlled ovarian stimulation for both parameters. These findings may relate to the hormonal status during ovarian controlled stimulation and the effects of higher serum estradiol. A significant decrease of all parameters for both groups was observed on the trigger day with rhCG, with the recovery of fluxometry parameters at embryo transfer day. The decrease of both resistance and pulsatility flow may be associated with the rhCG effect on vascularization, due to its up-regulation effect on vascular endothelial growth factors.

Subendometrial blood flow displayed a similar pattern with comparable values at baseline and increasingly higher values during stimulation and also a significant decrease after rhCG administration and recovery to previous values at embryo transfer. In a similar pattern values were statistical similar at baseline, and significantly different between the two groups afterwards.

The ratio obtained between subendometrial flow and uterine artery fluxometry parameters showed, for all parameters (resistance and pulsatility) and for both groups that values in the subendometrial compartment were sustained and lower in comparison to the uterine artery flow (values always under 1). This means that subendometrial territory has lower resistance to blood flow allowing further and privileged vascularization. We could also note that in terms of resistance index the values on the implantation group were lower with statistical difference between the two sets. In terms of pulsatility, values were also lower in comparison to uterine artery fluxometry, but no significant pattern was met. Since we are dealing with a reason between two values (pulsatility index from subendometrial flow and uterine artery), with increasingly higher values until trigger day with rhCG, followed by a significant drop and recovery afterwards at embryo transfer day, this might be an explanation to the observed pattern [17].

We could not refrain to uphold expectation of these results as they show a serial of values, demonstrating a certain pattern of evolution that one should expect from a transforming living tissue and its natural adaptations in need to further assist on the complex binding process of implantation.

## Conclusions

Endometrial receptivity plays an important role in the successful outcome in ART cycles. Much has improved over recent years in the area of embryo transfer, and embryo cultures. Yet the underlying mechanism that results in failure of implantation of a good quality embryo on a supposed receptive endometrium is still unclear. Implantation window is the most critical period of time in human reproduction. In a clinical point of view, practitioners need to have some objective measurements to determine the probability for a healthy pregnancy.

Many techniques have been developed but results are still controversial, or in some cases proven to be too invasive and lacking reliability especially in women with irregular menstrual cycles.

The continuous evolution of endometrium makes it difficult to establish a pattern that might be useful in identifying a receptive endometrium.

Ultrasound developments have been able to clarify and make aware more information about the morphokynetics of this tissue and its changes throughout the cycle. Better understanding of the role that makes an endometrium receptive may be the key in solving these issues, providing a diagnostic tool that will enhance ART cycles and elective embryo transfers more effective in producing better outcomes.

This study showed that endometrial 2D power doppler analysis may identify a receptive endometrium as soon as day 6 of ovarian stimulation. Uterine artery fluxometry and subendometrial blood flow as single evaluation parameters, or in combination as a ratio show a clear continuous mechanism that enables endometrium to become receptive to a healthy embryo. In this way clinicians may be made aware of this possibility and further enhance its procedures with better knowledge weather or not to perform embryo transfer on that given cycle.

## Data Availability

Encrypted non-disclosure data available at Open Science Framework database for peer review purpose only. Project name Physical Biomarkers in Endometrial Receptivity with access link: https://osf.io/hr25m/?view_only=8d5f6dcb8b25420bbd9188382163e7d7
